# Transcriptional Misexpression in Hybrids between Species Linked by Gene Flow Is Associated With Patterns of Sequence Divergence

**DOI:** 10.1093/gbe/evad071

**Published:** 2023-05-08

**Authors:** Fernando Díaz, Jason Wolf, Reinaldo A de Brito

**Affiliations:** Department of Biology, Colgate University, Hamilton, New York, USA; Milner Centre for Evolution and Department of Life Sciences, University of Bath, Bath, United Kingdom; Departamento de Genética e Evolução, Universidade Federal de São Carlos, São Carlos, Brazil

**Keywords:** hybrid incompatibility, *cis/trans* regulation, transgressive expression, introgression, speciation with gene flow, RNA-seq

## Abstract

The extent to which hybridization disrupts a gene's pattern of expression likely governs its propensity for introgression, whereas its extent of molecular divergence can itself underlie such disruption. Together, these phenomena shape the landscape of sequence and transcriptional divergence across the genome as species diverge. To understand this process, we characterize gene expression inheritance, regulatory divergence, and molecular divergence in the reproductive transcriptomes of species linked by gene flow: the fruit flies *Anastrepha fraterculus* and *A. obliqua*, which show evidence of gene flow despite clear evolutionary divergence. We find that their transcriptional patterns are a mosaic between those typically observed within and between allopatric species. Transcripts showing *transgressive* expression in hybrids or *cis*-regulatory divergence between species are associated with greater sequence divergence. This may reflect pleiotropic constraints that make them resistant to gene flow or they may be more likely to experience divergent selection. Although these more divergent gene classes are likely to be important contributors to species differences, they are relatively rare. Instead, most differentially regulated transcripts, including those linked to reproduction, show high degrees of *dominance* in hybrids and *trans*-regulated divergence between species, suggesting widespread genetic compatibility that potentially allowed for introgression. These findings provide insights into how postzygotic isolating mechanisms might evolve in the presence of gene flow: regions showing *cis*-regulatory divergence or *transgressive* expression contribute to reproductive isolation, whereas regions with *dominant* expression and *trans*-regulatory divergence allow for introgression. These patterns create a genomic mosaic of transcriptional regulation that is tied to sequence divergence.

**Significance Statement**

Divergence in gene expression regulation between species can impose a barrier to hybridization. Nonetheless, many species pairs experience substantial gene flow, often appearing as a mosaic of highly and lowly divergent genomic regions. To understand gene expression regulation and molecular divergence in such systems, we characterize transcription and its link to sequence divergence in hybrids of species connected by gene flow. We find that transcriptional patterns are a mixture of those typically observed within and between allopatric species. Although most transcripts exhibit regulatory patterns unlikely to be disruptive in hybrids, which presumably allows for introgression, those that exhibit disruptive patterns show greater molecular divergence. These findings provide important insights into how transcriptional regulation may shape gene flow.

## Introduction

Although species were once considered to be well-defined evolutionarily independent lineages, there is increasing evidence that there is a continuum of reproductive isolation and, hence, genetic exchange (Mallet et al. 2007; Martin et al. 2013; Harrison and Larson 2014). As a result, the genomes of what has been assumed to be well-defined divergent species often have been found to be mosaics, containing both a subset of regions homogenized by introgressive hybridization (Wu 2001) and a subset of divergent regions, which are presumably responsible for the genetic isolation (Harrison and Larson 2014). Although these “genomic islands” are well documented across different species and their molecular evolution has received considerable attention (Harr 2006; Mallet et al. 2007; Malinsky et al. 2015; Choi et al. 2020), their causes are still in debate (Kulathinal et al. 2009; Cruickshank and Hahn 2014; Han et al. 2017). The propensity for introgression and the extent of molecular divergence that shape the pattern of these islands are presumably governed by the array of phenomena that ultimately result in genetic incompatibilities, including transgressive phenotypes, genomic rearrangements, altered protein interactions, patterns of gene expression, and disrupted epigenetic mechanisms (Ortiz-Barrientos et al. 2006; Barbash 2010; Maheshwari and Barbash 2011). Individual genes and potentially larger genomic blocks can vary in their contribution to these phenomena and hence will vary in their propensity for introgression and degree of divergence. Those regions that remain compatible are able to introgress, potentially counteracting divergence by selection and drift (Mallet et al. 2007; Martin and Jiggins 2017; Nadeau and Kawakami 2018), whereas selection on regions associated with transcriptional misexpression and/or disrupted phenotypes in hybrids will contribute to maintaining or fostering divergence.

Transcriptional misexpression in hybrids is a consequence of the altered molecular interactions between alleles from the parental species (Signor and Nuzhdin 2018; Renaut et al. 2009; Wittkopp et al. 2004; Gordon and Ruvinsky 2012; Ortiz-Barrientos et al. 2006), suggesting that the mosaic of divergence can be, at least in part, understood through characterization of patterns of hybrid gene expression. Genes in regions showing recent or ongoing gene flow across species would presumably show patterns of expression consistent with genetic compatibility, whereas genes in regions resistant to gene flow would presumably show patterns of expression disruption that could underlie genetic incompatibilities. For example, individual genes (and their regulatory regions) can show sequence divergence that causes *cis*-regulatory differences (Morris et al. 2004; Barrière and Ruvinsky 2014; Wei and Zhang 2018). As a result, genes with divergent *cis*-regulatory sequences may be resistant to introgression because they maintain their divergent expression profile, leading to allelic imbalance in hybrids (Landry et al. 2005; Coolon et al. 2014; Signor and Nuzhdin 2018). Moreover, genes with divergent transcription factors between species show *trans-*regulatory divergence, where alleles from both species may respond similarly to the same transcription factors in hybrids. Therefore, divergence between species in transcription factors can lead to divergence in expression, but alleles can adopt the expression profile dictated by the transcription factors, which could facilitate introgression. This idea is supported by studies that, at a gross scale, suggest that *cis*-regulatory mechanisms are particularly associated with highly divergent genomes, such as those from interspecific crosses (Wittkopp et al. 2008; Signor and Nuzhdin 2018), whereas *trans*-regulatory mechanisms are more likely to account for expression divergence (ED) between genomes with low divergence, such as intraspecific crosses (Bell et al. 2013; Suvorov et al. 2013; Signor and Nuzhdin 2018).

Most intra- and interspecific patterns of gene regulation reflect the broader patterns of regulatory element evolution between species and are typically addressed by studying allopatric species. Although the regulation and inheritance of gene expression in hybrids are likely key factors in shaping the pattern of divergence across genomes, the relationship between these phenomena and molecular divergence in species showing isolation with gene flow remains poorly understood (Graze et al. 2012; Meiklejohn et al. 2014). Available evidence, particularly from closely related *Drosophila* species (Coolon et al. 2014; Meiklejohn et al. 2014; Banho et al. 2021), indicates that regulatory divergence in recently established species represents an intermediate mosaic with respect to that seen within species and between highly divergent species. However, the role of introgression in shaping the landscape of transcriptional misexpression in hybrids and regulatory divergence between recently diverged species remains unresolved (Martin and Jiggins 2017).

To understand the relationship between regulation and inheritance of gene expression in hybrids and patterns of sequence divergence between species, we use a pair of neotropical fruit fly species, *Anastrepha fraterculus* and *A. obliqua*, that diverged ∼2.6 Ma but still show strong signatures of recent gene flow (Díaz et al. 2018). These species belong to the *fraterculus* group, which harbors some of the most important agricultural pests in South America (Aluja 1994). Despite the presence of gene flow, these species can easily be distinguished by several phenotypic differences and show differences in ecologically relevant traits such as host preferences and reproductive behavior (Aluja 1994; Aluja et al. 1999; Sivinski et al. 1999). Detected gene flow suggests that prezygotic barriers to reproduction are leaky (Scally et al. 2016; Díaz et al. 2018). Indeed, hybrids between these species can be obtained in the laboratory, but postzygotic incompatibilities follow Haldane's rule when crossing *A. obliqua* females with *A. fraterculus* males, meaning that the offsprings of the heterogametic sex, in this case males (Selivon et al. 2005), are not viable (Selivon et al. 1999; Santos et al. 2001; Rull et al. 2018). Because genes associated with reproductive isolation are likely to evolve rapidly despite gene flow, we focus on transcriptomes of reproductive tissues, where patterns of ED are likely to be more detectable (Andrés et al. 2008). We compare transcriptional patterns of the parental species and their F_1_ hybrids to characterize patterns of expression inheritance (e.g., *additive*/*transgressive*) and regulatory divergence (e.g., *cis*/*trans*). We then relate these patterns to the degree of sequence divergence and gene flow to gain insights into how postzygotic isolating mechanisms might evolve in the presence of gene flow, while buffering against complete reproductive breakdown to facilitate introgression.

## Results

### Experimental Design

We performed crosses within and between the species *A. fraterculus* and *A. obliqua* and sampled male and female reproductive tissues from the two parental species and their hybrids. By sequencing transcriptomes from these samples, we characterized gene expression inheritance and regulatory divergence, and the degree of molecular divergence at these transcripts. We refer to the reciprocal hybrids as OF and FO (F = *A. fraterculus* and O = *A. obliqua*, where the female parent is listed first). Because the *♀ A. fraterculus* × *♂ A. obliqua* cross only produces female progeny (Santos et al. 2001), our analysis is restricted to three of the four classes of hybrids. Three biological replicates were performed per cross, which generated six samples for each parental species and OF hybrids and three for FO hybrids (only females), for a total of 21 RNA-Seq libraries.

### Sequencing and Trimming De Novo Assembly

Nearly 300 million paired-end read sequences were obtained from Illumina HiSeq runs, ranging from 11 to 24 million raw paired-end reads for each sample (supplementary table S1, Supplementary Material online). Because there is no available reference genome for *Anastrepha* species, we used Trinity software to assemble transcriptomes de novo. To minimize issues associated with redundancy and chimerism, we performed separate assemblies for male and female reproductive transcriptomes. In addition, we used a cleaning assembly strategy based on the software set Trinity-Bowtie2-RSEM-CD-HIT-EST as recommended by Yang and Smith (2013), to reduce redundant and chimeric sequences, while maximizing the number of truly representative isoforms. The generated transcripts were filtered by abundance, keeping only transcripts with expression in both species while collapsing transcripts with highly similar sequences. These transcriptomes contained between 80 and 100 thousand transcripts per reproductive library.

Our analysis of regulatory divergence involves allele-specific expression (ASE), which relies on the identification of the parental origin of reads sequenced in hybrid samples. To account for potential species-mapping bias to the reference, we generated a “common diploid *Anastrepha* reference” while keeping only transcripts with fixed divergent single nucleotide polymorphisms (SNPs) between the species. All reads were then mapped back to this reference using SNP-tolerant alignment as implemented in genomic short-read nucleotide alignment program (GSNAP) (Wu and Nacu 2010) to ensure that reads coming from both species map with equal probabilities. From these assembled transcripts, after a final filtering of 3 cpm in at least two replicates from each profile, we detected at least one fixed SNP between the parental species in 10,628 and 9,709 transcripts for males and females, respectively.

The distribution of fixed SNPs followed similar distributions in male and female transcriptomes, with a median of four fixed SNPs per transcript between the species (supplementary fig. S1, Supplementary Material online). Hybrids between these species exhibited a nearly 1:1 ratio of mapping reads based on their parental origins (supplementary table S2, Supplementary Material online) as expected in the absence of mapping bias. We then confirmed these expectations by simulating reads extracted from both assemblies (*A. fraterculus* and *A. obliqua* derived references) in the common diploid *Anastrepha* genome. The same number of reads was simulated for each polymorphic site and then mapped back to the reference; transcripts in all samples followed an expression ratio of 1 (supplementary fig. S2, Supplementary Material online). Following read mapping, we created a read count matrix for the parental species and hybrids. Because the number of reads in the dataset was restricted to those unambiguously assigned to their parental origins in hybrids, we performed a power analysis to detect significant differential gene expression with the filtered dataset using the R package “*Proper*” (Wu et al. 2015). We performed 20 different simulations of RNA-Seq using the same number of replicates, transcripts, and coverage obtained after filtering our data for reads overlapping fixed SNPs between the species (supplementary fig. S3, Supplementary Material online). We then assessed the power to detect significant differential expression using different log_2_FC thresholds under a common false discovery rate (FDR) of 0.05. We found that the power to detect differential gene expression is maximized and approximates the canonical 80% when using a threshold of log_2_FC > 1.25, which is a standard threshold used in other ASE studies (Bell et al. 2013). Based on these results, these count data and defined thresholds were used for all our downstream analyses.

Another potential bias that may be related to the ASE framework is the fact that the dataset is restricted to reads overlapping fixed divergent SNPs between the species. This may bias the results increasing the coverage and therefore the power to detect differential expression toward those transcripts with a higher number of fixed SNPs. Although the number of fixed SNPs is indeed correlated with coverage, transcripts with higher coverage are not more likely to show significant ED between species or ASE in hybrids. Moreover, the average coverage of ED transcripts was in fact lower that of no-ED transcripts in both males and females (mean logCPM = 4.6 and 4.9 for ED and no-ED transcripts in females, respectively, and logCPM = 4.3 and 4.6 in males), indicating that higher coverage does not lead to biased result of gene expression.

### Expression Divergence Between *A. fraterculus* and *A. obliqua*

We scored significant gene ED using the log_2_FC between species following an FDR correction of 0.05 (Benjamini and Hochberg 1995) and a log_2_FC threshold of > 1.25. Using these thresholds, the number of transcripts with significant ED between species was substantially different between reproductive transcriptomes of males and females: 2,248 female reproductive transcripts (23%) exhibited ED between species, whereas 1,177 ED transcripts (11%) were detected in males (supplementary table S3, Supplementary Material online and supplementary fig. S4, Supplementary Material online).

### ASE in Hybrids

Next, we analyzed ASE patterns in the hybrids by classifying reads according to their parental alleles using species-specific SNPs. We scored significant ASE using the log_2_FC of expression between alleles in hybrids following an FDR correction of 0.05 and a log_2_FC threshold of > 1.25. OF and FO hybrid females had 10% and 11% of filtered transcripts showing ASE, whereas FO males exhibited 14% of transcripts with ASE (supplementary table S4, Supplementary Material online and supplementary fig. S4, Supplementary Material online). These differences between reciprocal crosses were, however, not observed when comparing the inheritance mode or regulatory divergence of their gene expression (see below).

### Modes of Gene Expression Inheritance

**Fig. 1:**
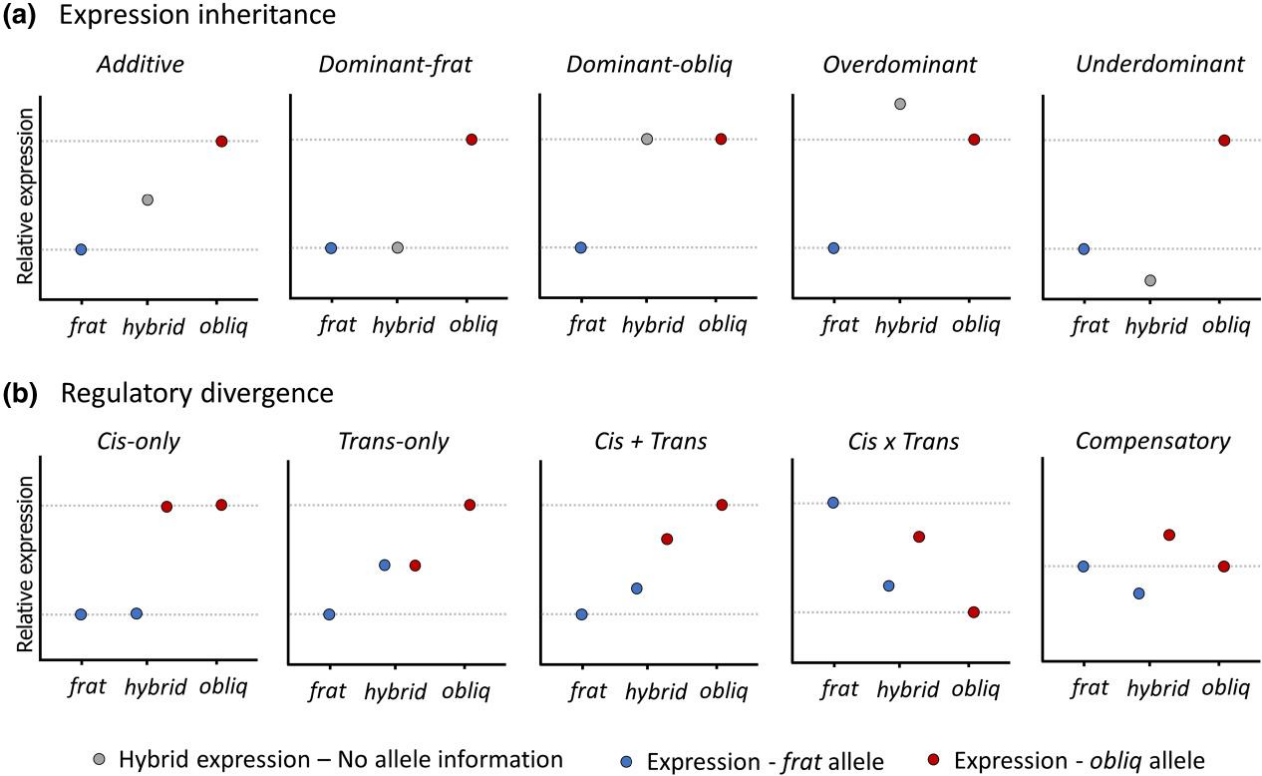
Schematic representation of potential examples of gene expression inheritance and patterns of regulatory divergence (*cis* and *trans*) between *A. fraterculus* (*frat*) and *A. obliqua* (*obliq*). a) The relative expression of transcripts in hybrids and ED between the parental species determines the classification of individual transcripts as: *additive*, *dominant* (*frat* and *obliq*), *overdominant*, and *underdominan*t. b) The relative allele-specific expression (ASE) in hybrids and ED determines the mechanisms of regulatory divergence: *Cis-only, Trans-only, Cis* + *Trans, Cis × Trans, and Compensatory*. Because alleles in hybrids are exposed to the same genetic background, ASE in hybrids represents the level of ED due to *cis*-acting regulation, whereas the *trans-*acting component can be extracted from total ED between species in comparison with the ASE in hybrids. Note that these plots only represent examples designed to capture the basic properties of each scenario (i.e., alternative patterns of gene expression that can potentially match each scenario).

**Fig. 2:**
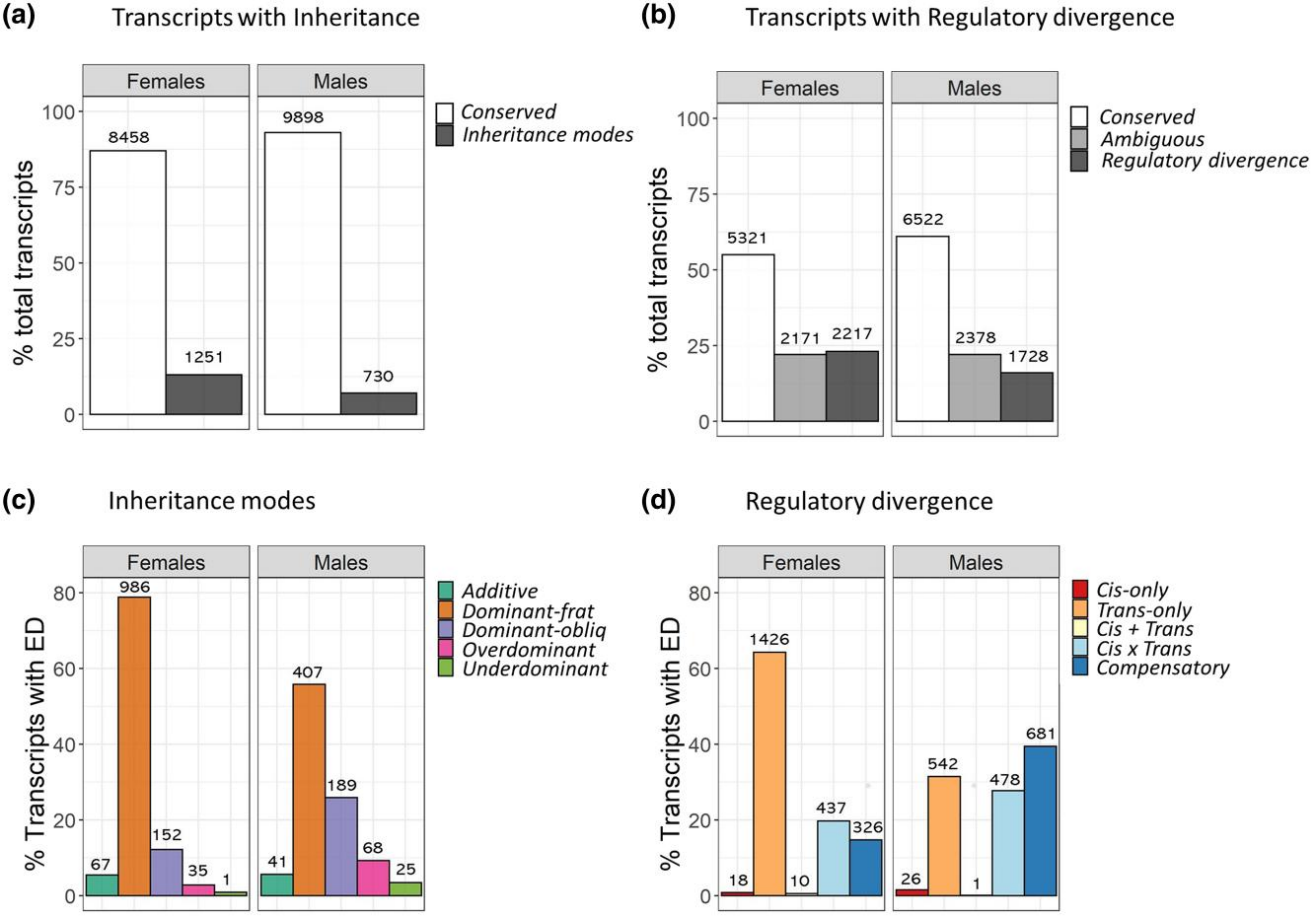
Classification of transcripts according to their mode of gene expression inheritance and mechanisms of regulatory divergence. Barplots show the number and percentage of total transcripts whose gene expression was classified into categories of a) expression inheritance and b) regulatory divergence. After excluding *conserved* and *ambiguous* transcripts (in the case of regulatory categories), the relative distribution of transcripts with ED (ED between the parental species) is shown across the five categories of c) inheritance modes and d) regulatory divergence. Note that these panels provide a breakdown of the distribution of transcripts with significant ED and hence do not include the conserved or ambiguous classes. The number of transcripts in each category is indicated on top of each bar.

We classified transcripts that showed significant ED between parental species and according to their inheritance modes of gene expression in hybrids (fig. 1*a*) (based on log_2_FC) following McManus et al. (2010) and Bell et al. (2013). Transcripts were classified into five categories of expression inheritance: *additive*, *dominant* (*dominant-frat* and *dominant-obliq*, to differentiate between the dominance of *A. fraterculus* and *A. obliqua*, respectively), *transgressive* (*overdominant* and *underdominant*), or as *conserved* when no significant differential expression was detected (supplementary fig. S5, Supplementary Material online, see also Materials and Methods). Transcripts with no evidence of ED between parental species or that their expression was not significantly different from that in hybrids (accounting for 85–93% of transcripts) were classified as *conserved* (fig. 2*a* and supplementary table S4, Supplementary Material online).

We used a generalized linear model (GLM) analysis to investigate differences in the distribution of the remaining transcripts across categories between male and female reproductive tissues (supplementary table S5, Supplementary Material online). For this, we used categories of inheritance, sex, and reciprocal cross type (OF and FO) as independent variables, with the number of transcripts detected as the dependent variable. After controlling for library size, the relative number of transcripts detected showed a significant interaction effect between categories of inheritance and sex (supplementary table S5, Supplementary Material online). The interaction effect indicates that the number of transcripts, or their distribution across categories, is different between males and females (fig. 2*c*). The number of detected transcripts was greater for females than males across categories (fig. 2*c*). The majority of transcripts show dominance (more often of *A. fraterculus* over *A. obliqua*), followed by the *transgressive* categories, with *overdominance* being significantly more common than *underdominance* (fig. 2*c*). The direction of the cross (e.g., FO or OF) did not significantly affect the number of transcripts detected under these categories.

### Regulatory Divergence Inferred From ASE

Patterns of ED between the parental species and ASE in hybrids were used to infer *cis* and *trans*-regulatory effects for each transcript based on significant log_2_FC estimates of differential gene expression (fig. 1*b*), following McManus et al. (2010) and Bell et al. (2013). We classified transcripts into five different patterns of regulatory divergence based on relative expression changes: *cis*-only, *trans*-only, *cis* + *trans, cis × trans*, and *compensatory* (supplementary fig. S6, Supplementary Material online, see Materials and Methods). Transcripts were classified as *conserved* when no significant differential expression was detected or as *ambiguous* when expression patterns did not follow clear expectations according to these criteria. We found that between 55% and 61% of transcripts were *conserved* (fig. 2*b* and supplementary table S4, Supplementary Material online), showing no ED between species and no significant ASE in hybrids, whereas about 22% of transcripts were classified as *ambiguous* (fig. 2*b*). The remaining transcripts were classified under five regulatory categories, and their distribution across profiles was compared using a GLM analysis (supplementary table S5, Supplementary Material online).

After controlling for library size, the relative number of transcripts detected was significantly influenced by regulatory categories, sex, and their interactions (supplementary table S5, Supplementary Material online). The number of transcripts was substantially different across categories (fig. 2*d*), with the majority of transcripts showing *trans*-only effects and interactions between *cis* and *trans* (*cis × trans* and *compensatory*), whereas only a few transcripts showed evidence for *cis*-only or *cis* + *trans*-regulatory effects (fig. 2*d*). The interaction effect indicates that the total number of transcripts or their distribution across categories is different between males and females (fig. 2*d*). The number of *trans-*only transcripts was greater in female than in male reproductive tissues. On the other hand, the number of transcripts with evidence for *cis* and *trans* interactions (*cis × trans* and *compensatory*) was higher for male transcriptomes (fig. 2*d*). The direction of the cross (e.g., FO or OF) did not significantly affect the number of transcripts detected under these categories (supplementary table S5, Supplementary Material online).

### Potential Contribution of X-linked Transcripts to the ASE Analysis

In the absence of a reference genome for *Anastrepha* species, transcripts cannot be assigned to specific chromosomes. As a result, X-linked transcripts could potentially be incorrectly classified as showing significant ASE in males because they necessarily show hemizygous expression and asymmetrical inheritance (i.e., X-linked transcripts in males produced by a particular direction of a cross come from the female parental species). Because this could bias estimates of regulatory divergence in males, we investigated the potential for X linkage to influence our results by using ASE patterns in hybrid males to identify transcripts showing expression consistent with X linkage. For this, we identified transcripts with significant allelic imbalance due to the complete absence of reads from the *A. fraterculus* allele in hybrid males, reflecting the fact that X-linked transcripts in males are inherited from *A. obliqua* (see Materials and Methods). We found 49 transcripts consistent with this pattern, distributed in four categories of regulatory divergence: *cis-only* (3 transcripts), cis*trans (10 transcripts), *compensatory* (21 transcripts), and *ambiguous* (15 transcripts). Although we cannot determine what proportion of these 49 transcripts are, in fact, on the X chromosome, the fact that this small proportion (1.2% of transcripts with regulatory divergence in males) represents the upper limit to their potential total contribution indicates that they are unlikely to introduce meaningful biases in our analysis. Because these transcripts cannot be unambiguously assigned to the X chromosome and represent such a tiny percentage of transcripts with regulatory divergence, they have not been removed from our analyses. However, their hemizygous expression could potentially introduce bias if it were to lead to a large proportion of these transcripts being incorrectly classified as *dominant-obliq* (because hybrid males would only express the *A. obliqua* allele for X-linked transcripts, cis-regulation of the loci would result in the hybrid matching the *A. obliqua* parental pattern). For example, such a bias could potentially explain the higher proportional representation of the *dominant-obliq* category within the set of transcripts that show nonconserved expression inheritance in males compared with females (fig. 2*c*). However, we find that only 5 of the 189 (2.6%) transcripts classified as *dominant-obliq* show an expression profile consistent with X linkage in males, which is the same number that shows *dominant-frat* inheritance, indicating that no such bias exists in our analysis. Thus, it appears that the presence of unidentified X-linked transcripts is unlikely to be an important source of bias in our findings.

### Expression Divergence across Categories of Inheritance and Regulatory Divergence

**Fig. 3:**
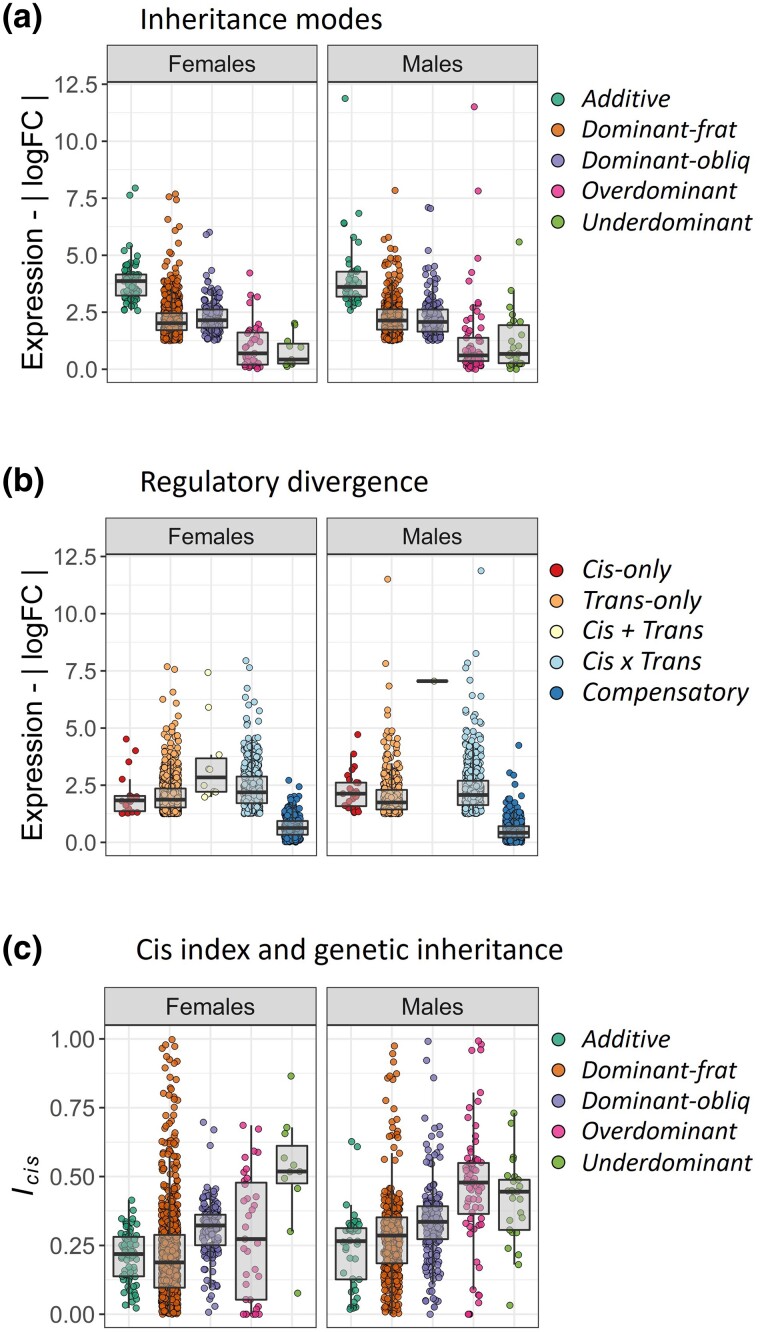
Patterns of ED across categories of genetic inheritance and gene regulation between *A. fraterculus* and *A. obliqua*. ED between the species was compared across a) modes of expression inheritance and b) regulatory divergence. Boxplots compare variation of absolute relative expression (a and b) between parental species (| log_2_FC *frat*/*obliq* |). c) The *cis* index *(I_cis_)* of regulatory divergence was compared across categories of genetic inheritance in hybrids. Boxplots compare the average variation of *cis* index per transcript.

The level of ED was substantial across inheritance categories (fig. 3*a* and supplementary table S5, Supplementary Material online) and regulatory mechanisms (fig. 3*b* and supplementary table S5, Supplementary Material online) between species. ED tended to be higher for *additive* transcripts, followed by *dominant* and, finally *transgressive* transcripts (*over* and *underdominant*) (fig. 3*a*). These results may, in part, simply reflect the classification process for gene expression inheritance because the hybrid expression is more likely to fall somewhere in between the expression values of the parental species when the two are very different. Similarly, in cases where the difference between the parental species is small, any expression difference in hybrids is more likely to place their expression outside the range of the parental species. On the other hand, ED was substantially higher for transcripts exhibiting *cis* + *trans* regulatory divergence, whereas the lowest ED was detected for transcripts in the *compensatory* category and no substantial differences across the remaining patterns (fig. 3*b*). The direction of the cross (e.g., FO or OF) did not significantly affect the level of ED detected under these categories (supplementary table S5, Supplementary Material online).

### Cis Index and Inheritance Modes

We compared the *cis* index of gene regulation (relative size of the *cis* to *trans* effect per transcript) across inheritance modes of expression (supplementary table S6, Supplementary Material online). The GLM results showed that the *cis* index was significantly different across categories of inheritance modes and sex, as well as their interactions (supplementary table S6, Supplementary Material online and fig. 3*c*). The *cis* index tended to be higher for *transgressive* transcripts (*over* and *underdominant*), followed by *dominant* transcripts (often higher in *dominant-obliq* than *dominant-frat*), and finally *additive* transcripts (fig. 3*c*). The direction of the cross (e.g., FO or OF) did not significantly affect the *cis* index detected across categories of expression inheritance (supplementary table S6, Supplementary Material online).

### Relationship between Molecular Divergence, Expression Divergence, and Allele Imbalance in Hybrids

**Fig. 4:**
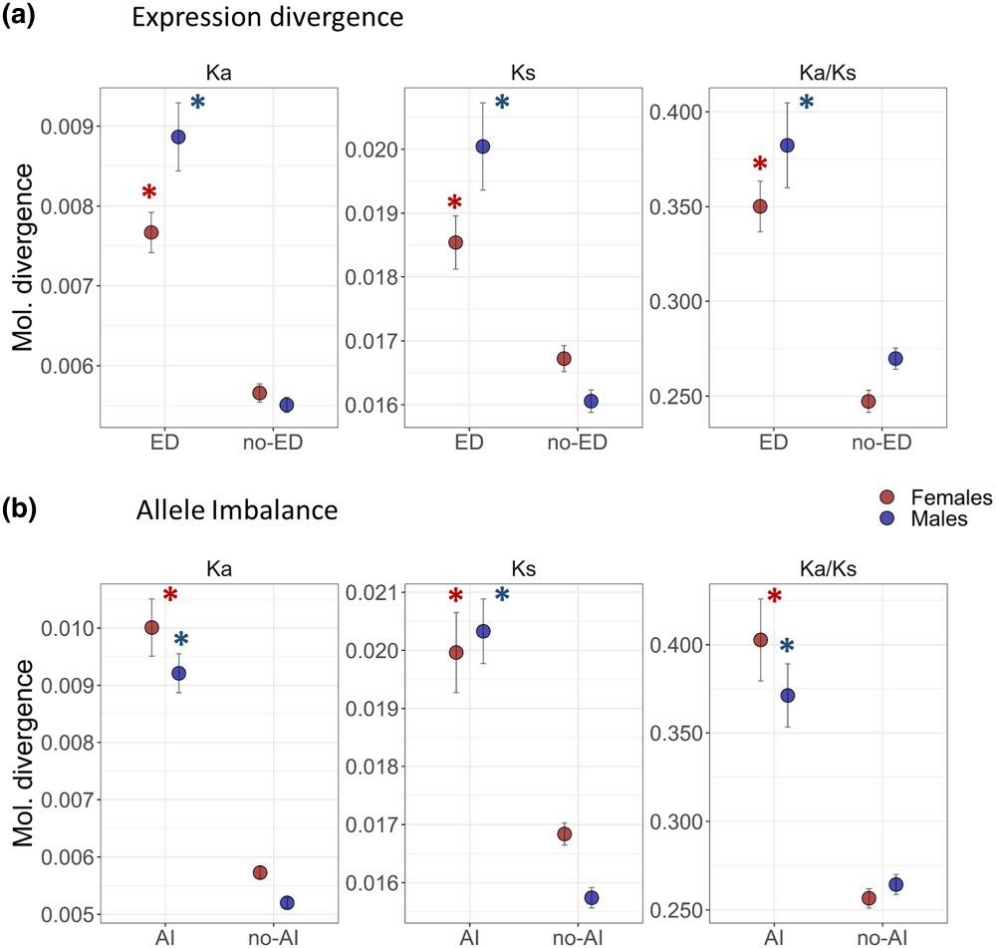
Patterns of molecular divergence associated with ED and allele imbalance in hybrids between *A. fraterculus* and *A. obliqua*. The plots compare the average molecular divergence (Ka, Ks, and Ka/Ks) for transcripts with significant a) ED and b) allele imbalance (AI). *Indicates significant comparisons with α = 0.05 following GLM analysis between groups for males and females.

We estimated molecular divergence based on the rate of synonymous (Ks) and nonsynonymous substitutions (Ka) and their ratio (Ka/Ks) for the entire dataset in male and female reproductive transcriptomes. Then, we compared molecular divergence in transcripts showing significant ED between species and allelic imbalance in hybrids (AI), with nonsignificant transcripts (no-ED and no-AI, respectively). The level of molecular divergence obtained from Ka, Ks, and Ka/Ks estimates followed similar patterns, being significantly higher for transcripts with significant ED between species when compared with no-ED transcripts (fig. 4*a*). The molecular divergence estimates for these three indices differed between male and female reproductive transcriptomes, with males exhibiting significantly higher values when compared with those of females. Similarly, molecular divergence estimates were consistently higher for transcripts with significant AI, but no significant differences between males and females were found (fig. 4*b*).

### Relationship between Molecular Divergence and Expression Inheritance in Hybrids

**Fig. 5:**
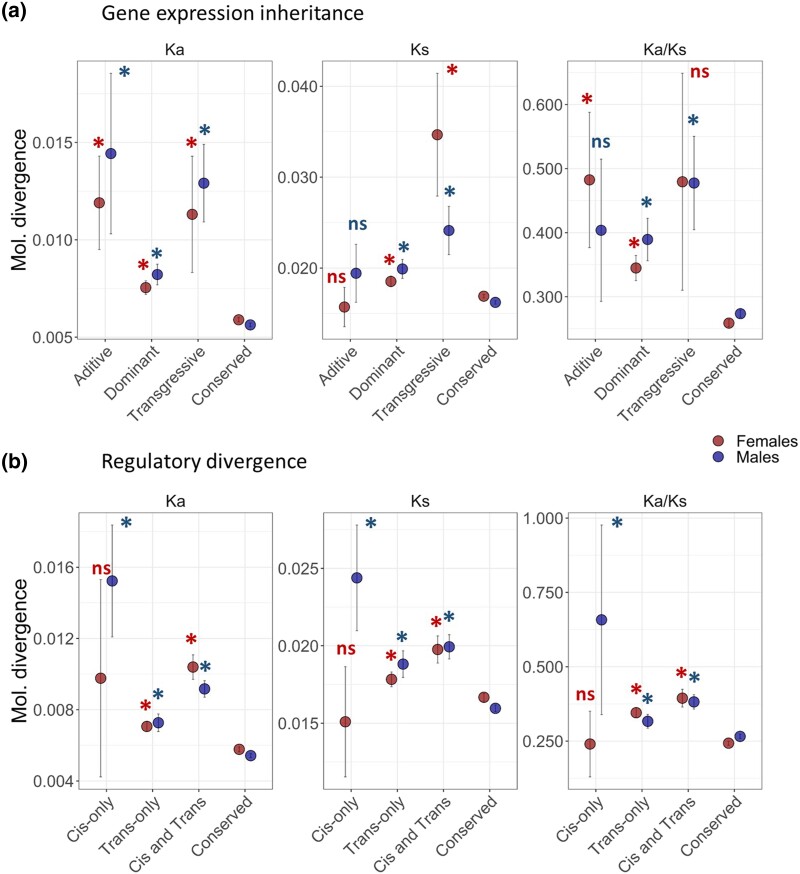
Patterns of molecular divergence associated with expression inheritance and regulatory divergence between *A. fraterculus* and *A. obliqua*. The plots compare the average molecular divergence (Ka, Ks, and Ka/Ks) across different classes of a) gene expression inheritance and b) regulatory divergence. *Indicates significant comparisons with α = 0.05 following GLM analysis for the comparison between each class and the conserved group of transcripts for males and females.

When comparing molecular divergence across categories of expression inheritance, we found that Ka and Ks estimates followed slightly different patterns. All categories of inheritance show significantly higher Ka divergence when compared with *conserved* transcripts, and *additive* transcripts show the highest levels of molecular divergence (fig. 5*a*). Likewise, *dominant* and *transgressive* transcripts showed significantly higher Ks divergence estimates, whereas Ks estimates from *additive* transcripts are not significantly different from *conserved* transcripts (fig. 5*a*). All categories have higher Ka/Ks ratios when compared with *conserved* transcripts, although male *additive* transcripts and female *transgressive* transcripts values are not significant, perhaps due to a large variance. Interestingly, *transgressive* transcripts show the highest molecular divergence for Ks values between males and females (fig. 5*a*).

### Relationship between Molecular and Regulatory Divergences

Estimates of molecular divergence are also different for categories of regulatory divergence, showing consistent patterns across Ka, Ks, and Ka/Ks ratios (fig. 5*b*). In this case, all regulatory divergence categories show significantly higher molecular divergence when compared with *conserved* transcripts. The only exception is that female *cis*-only transcripts are not significantly different from *conserved* transcripts, whereas male *cis*-only transcripts exhibit the highest molecular divergence (fig. 5*b*), even higher than that of *transgressive* transcripts (fig. 5*b*). These results suggest that transcriptional differences in expression and regulation between *Anastrepha* species correlate with patterns of molecular divergence.

### Functional Analysis

**Fig. 6:**
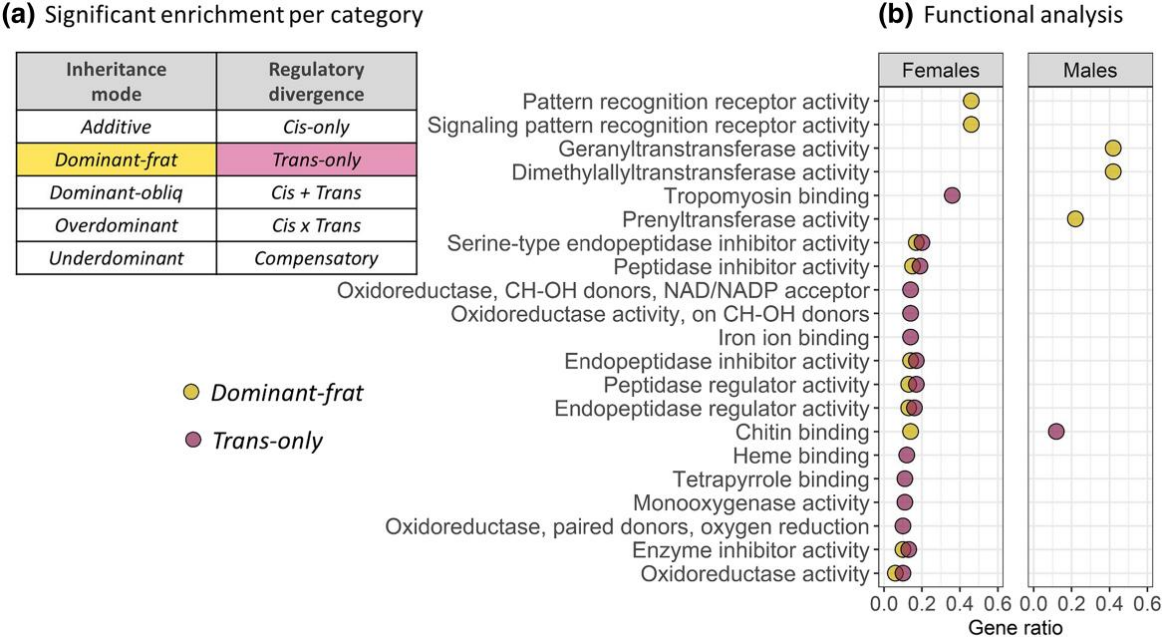
Functional analysis of male and female reproductive transcripts following modes of expression inheritance and regulatory divergence between *A. fraterculus* and *A. obliqua*. a) Gene ontology enrichments were significant for transcripts with *dominant-frat* expression in hybrids as well as those with *trans*-only regulatory divergence. b) Functional analysis indicates that these categories are enriched for regulatory networks associated with male and female sexual interactions in insects. Gene ratio of significantly detected transcripts within each enriched category is indicated (gene ratio = significant transcripts in category/total number of transcripts in category).

To analyze the functional pathways associated with gene expression inheritance and regulatory divergence between *A. fraterculus* and *A. obliqua*, we performed gene ontology (GO) enrichment analysis (fig. 6). We found evidence for functional specialization in the sets of transcripts showing *trans*-only divergence or *dominant-frat* expression in hybrids between these *Anastrepha* species (fig. 6*a*). Although the rest of the categories did not show significant gene enrichments in female transcriptomes, these two sets of transcripts were strongly associated with important functions previously linked to the female reproductive tract of numerous insect species (fig. 6*b*). These transcriptional changes are enriched for the following three groups of functional pathways (fig. 6*b*): 1) epithelial modifications, 2) protease/protease inhibitors, and 3) immune/defense response. Similarly, male transcriptomes were significantly enriched for at least three functional pathways that have been previously associated with male reproductive tissues.

## Discussion

Using patterns of transcriptional divergence and ASE in hybrids between species historically connected by gene flow (*A. fraterculus* and *A. obliqua*), we have been able to characterize regulatory divergence and expression inheritance and link these to sequence divergence. Expression inheritance characterizes the relationship between the pattern of gene expression in hybrids and that of the parental species, whereas regulatory divergence uses the patterns of ASE in hybrids to characterize the expression regulation of the gene copies coming from the two parental species. We find that transcriptional patterns in hybrids show a mosaic between those typically observed within and between allopatric species. A large proportion of the differentially expressed transcripts between the species show an interaction between *cis* and *trans* regulatory divergence, which is frequently seen in interspecific hybrids (Wittkopp et al. 2008; Coolon et al. 2014). There is also a high proportion of transcripts showing *trans*-only regulatory divergence and *dominance* expression inheritance, which is typically seen in within-species ASE studies (Wittkopp et al. 2008; Suvorov et al. 2013). The molecular evolution of differentially regulated transcripts is consistent with expectations from weak genetic incompatibilities between species experiencing substantial introgression (Díaz et al. 2018), where interspecific crosses can generate hybrids following a Haldane's rule causing male lethality in one of the crosses (Selivon et al. 1999; Santos et al. 2001; Rull et al. 2018).

### Gene-expression Inheritance in Hybrids

We detected all possible modes of expression inheritance, with a similar distribution across male and female reproductive transcriptomes. Most transcripts that are differentially expressed fall into one of the *dominant* categories (80–95%), followed by *transgressive* (*over-* or *underdominant*) and *additive* modes of inheritance (2–6%). *Transgressive* transcripts were not as common as described in other interspecific hybrids (Landry et al. 2007; Wittkopp et al. 2008) (see supplementary table S7, Supplementary Material online with comparisons across different studies). Indeed, the proportion of *transgressive* transcripts (3–13%) is over three times lower than that observed in interspecific *Drosophila* hybrids (35–69%) (Ranz et al. 2004; McManus et al. 2010) and even lower than what was found in some intraspecific crosses (Bell et al. 2013). In contrast, more distantly related species, such as *Caenorhabditis briggsae* and *C. nigoni*, have a high proportion of transgressively expressed genes, as well as *compensatory* changes (Sanchez-Ramirez et al. 2021). Transgressive phenotypes are often assumed to be a consequence of *transgressive* expression (Renaut et al. 2009; Rieseberg et al. 2003; Landry et al. 2005; Swanson-Wagner et al. 2006), and hence, the fact that we see very few transcripts with *transgressive* expression in hybrids (compared with nontransgressively inherited transcripts) is consistent with the observation that lab generated hybrids are viable, fertile, and show no visible abnormalities aside from the inviability of males in one of the interspecific crosses, which follows Haldane's rule (Selivon et al. 1999; Santos et al. 2001; Rull et al. 2018). Those transcripts that do show *transgressive* expression generally show low ED, which may simply reflect the fact that any expression difference in hybrids is, therefore, likely to place them outside the range of the parental species. In contrast, transcripts showing *additive* inheritance generally exhibit the highest ED, which may reflect the fact that hybrids are more likely to fall somewhere within the large gap between the expression values of the parental species.

### Regulatory Divergence

Although the number of studies evaluating ASE in hybrids between species is limited, comparisons across *Drosophila* (Coolon et al. 2014; Meiklejohn et al. 2014) and plant species (Zhang et al. 2019) have shown that the proportion of transcripts with *cis* or *trans* regulatory divergence can change with the level of species divergence (see supplementary table S7, Supplementary Material online with comparisons across different studies). Recently diverged species (or populations within species) tend to be recently diverged species (or populations within species) are dominated by *trans* regulation, whereas highly divergent species show higher proportions of genes under *cis* regulatory divergence (Wittkopp et al. 2008; Coolon et al. 2014). This suggests that *trans* regulatory changes accumulate more rapidly than divergence at *cis* elements (or at least accumulate earlier in the process of divergence). The mechanisms underlying this pattern, or how widespread it is across species, are still unclear. The most accepted hypothesis suggests that divergence at *cis* elements evolves more slowly because it results from accumulated changes at individual genes that are independent of the background genome (Coolon et al. 2014; Signor and Nuzhdin 2018) (meaning each gene showing *cis* regulatory divergence potentially reflects an independent evolutionary episode). In contrast, a single episode of mutation and evolution at a *trans* regulator (i.e., a transcription factor) can potentially cascade across multiple target genes (Wittkopp et al. 2008; Mack and Nachman 2017), leading to ED at many genes all at once, which potentially explains the higher number of *trans* regulated genes. However, because these impacts on the expression of multiple genes, *trans* regulators are also likely constrained by their pleiotropic effects (which could amplify the effects of mutations on fitness). Therefore, the overall contribution of *trans* regulation to species divergence likely depends on the nature and extent of pleiotropic interactions between *trans* regulators and their target genes.

Transcripts with ED showing *cis*-*trans* interactions (i.e., *cis × trans* or *compensatory*) account for 67% and 34% of transcripts for males and females, respectively. These transcripts show opposing effects on gene expression, with a large proportion of transcripts showing *compensatory* regulation. This pattern, along with the high proportion of transcripts with *conserved* expression (over 83%), strongly indicates the presence of evolutionary constraints on gene expression (Graze et al. 2012; Meiklejohn et al. 2014; Chen et al. 2015; Signor and Nuzhdin 2018) in a substantial number of transcripts. This general pattern is widespread, being also found in yeast, worms, *Drosophila*, and mice (Mack and Nachman 2017; Signor and Nuzhdin 2018) and has been explained by coadaptation of regulatory elements mediated by stabilizing selection of gene expression. Despite the extensive evidence for this pattern, its evolutionary significance and the relative contribution of selection or parallel evolution are usually unexplored (Signor and Nuzhdin 2018). Existing models and theoretical considerations suggest that independent evolution of *trans* regulators must arise following speciation as a result of their large pleiotropic effects on their target genes (Ortiz-Barrientos et al. 2006; Signor and Nuzhdin 2018). Then, stabilizing selection on gene expression favors compensation mediated by *cis* elements on the target genes (Ortiz-Barrientos et al. 2006; Yoo et al. 2013; Fear et al. 2016; Mack and Nachman 2017; Signor and Nuzhdin 2018).

Because the cross between *A. obliqua* females and *A. fraterculus* males does not produce any male offspring, hybrid males only possess *A. obliqua* alleles at any X-linked *trans*-regulatory factors, which could potentially influence the observed patterns of expression inheritance or regulatory divergence in males. For example, hybrid males would have the same X-linked *trans*-regulatory factors as the *A. obliqua* parental line, which could lead to the appearance of *dominant-obliq* inheritance in males if such factors play an important role in controlling broad expression patterns. This effect could, therefore, potentially contribute to the higher proportional representation of *dominant-obliq* inheritance in males (26% of the transcripts with nonconserved inheritance, fig. 2*c*) relative to females in the same cross (12% of the transcripts with nonconserved inheritance, fig. 2*c*). It is important to recognize, however, that this scenario does not represent a bias in the estimation of gene expression inheritance in males in this cross. Rather, the imbalance of X-linked regulatory factors in the heterogametic hybrid simply reflects the biological basis of differential gene expression regulation in males and females, which is expected in any system with XY sex determination. The presence of Haldane's rule in only one direction of the interspecific cross, however, does impose a biological constraint on the experimental design that means that the estimated patterns of expression inheritance and transcriptional regulation in males cannot be compared with the opposite direction of the cross. Therefore, the causes and consequences of gene expression inheritance and regulatory divergence in males should be interpreted with the potential influence of X-linked *trans*-regulatory factors in mind.

### Transcriptional Misexpression and Sequence Divergence

We found a significant association between rates of molecular evolution of individual transcripts and their level of ED between species, as well as with the extent of allelic imbalance in hybrids. These results support the hypothesis that protein sequence and ED are influenced by similar selective processes (Lemos et al. 2005; Go and Civetta 2020). Within these transcripts, we discovered that transcripts associated with *transgressive* expression and those showing *cis*-only regulatory divergence exhibit the greatest molecular divergence. More interestingly, these groups of transcripts also show the highest difference in patterns of molecular evolution between sexes, with males showing higher evolutionary rates than females. These two groups of transcripts represent outliers in the distribution of evolutionary rates across the whole genome. *Transgressive* female transcripts exhibit the highest level of divergence at synonymous sites, whereas male *cis*-regulated transcripts had the highest Ka/Ks divergence. Additional genomic features may correlate with sequence divergence, such as gene length and absolute expression values (Nuzhdin et al. 2004; Lemos et al. 2005). However, molecular evolution at outliers might provide insights into the role played by selection (Osada et al. 2017; Go and Civetta 2020; Del Amparo et al. 2021; Sanchez-Ramirez et al. 2021) and introgression in these two sets of transcripts.

In females, *transgressive* transcripts exhibit a substantial number of synonymous substitutions. The recent divergence between *Anastrepha* species (∼ 2.6 Ma) suggests that synonymous sites are not saturated, and therefore our estimations are likely reflecting the influence of genetic drift and/or introgression (Díaz et al. 2018; Congrains et al. 2021). The effect of genetic drift is widespread, whereas introgression varies across the genome, which suggests lower introgression of *transgressive* female transcripts. These transcripts do not show an elevated Ka/Ks ratio (i.e., are not significantly elevated over that of *conserved* transcripts), indicating that these transcripts are not experiencing recurrent divergent positive selection. The rates of molecular evolution in *cis*-regulated female transcripts are not significantly different from those of *conserved* transcripts (i.e., Ka, Ks, and Ka/Ks). On the other hand, male transcripts exhibited an elevated Ka/Ks in both *transgressive* and *cis*-regulated transcripts, with the later showing the highest Ka/Ks estimates across the genome, which indicates a more relevant role of positive or relaxed selection in males (Nuzhdin et al. 2004; Lemos et al. 2005).

The patterns of molecular evolution in *transgressive* and *cis*-regulated transcripts are consistent with the fact that these two groups of transcripts are associated with hybrid misexpression and are likely to influence hybrid fitness (Renaut et al. 2009; Sanchez-Ramirez et al. 2021). Although *transgressive* transcripts are outside the gene expression range in the parental species, transcripts under *cis* regulation result from significant ASE because allelic differences at *cis* elements are the only source of regulation in hybrids (i.e., their regulatory background is the same for both alleles). Our estimations of I_cis_ (i.e., the *cis* index, or relative contribution of *cis-* over *trans*-regulation for individual transcripts) further support this hypothesis, because *transgressive* transcripts also had the highest level of *cis* regulation. This is consistent with previous research and models of hybrid incompatibility (Coyne and Orr 1997; Landry et al. 2005; Masly and Presgraves 2007; Ortiz-Barrientos et al. 2006; Presgraves 2008; Maheshwari and Barbash 2011; Cattani and Presgraves 2012; Turelli et al. 2014; Lopez-Maestre et al. 2017).

*Transgressive*- and *cis-regulated* transcripts also represent the smallest fraction of transcriptional differentiation between *Anastrepha* species. Although most of the transcriptional landscape is dominated by transcripts that do not show interspecific differences, those that differ tend to show a pattern of *compensatory (males), trans-only* regulation (females), and *dominant* inheritance in hybrids. This is consistent for species that diverged recently and still experience gene flow, because their transcriptional incompatibilities are weak enough to allow hybrids to survive and reproduce in order to account for the presence of introgression between the species (Díaz et al. 2018).

Despite substantial gene flow previously reported between these species in both directions (Scally et al. 2016; Díaz et al. 2018; Congrains et al. 2021), we detected a preponderance of *dominance* in the expression of *A. fraterculus* over *A. obliqua* transcripts. The underlying causes of this result are still unclear, but a similar pattern of dominance from one species over another has been observed in several different groups of organisms, from polyploids in plants, to a number of insect species (McManus et al. 2010; Yoo et al. 2013; Edger et al. 2017; Sanchez-Ramirez et al. 2021; Zhang et al. 2021). Functional analyses of GO categories of these transcripts indicates that *dominant-frat* and *trans*-only transcripts are enriched for regulatory networks that have been associated multiple times with the female postmating response in insects (Mack et al. 2006; Kocher et al. 2008; Alfonso-Parra et al. 2016; Al-Wathiqui et al. 2016; Thailayil et al. 2018; Fowler et al. 2019; Liu and Hao 2019; Gao et al. 2020). Similar studies in males detected that these same categories are enriched for regulatory networks associated with spermatogenesis (Dugan and Allen 1995; Adolphsen et al. 2012). These results likely show that female (e.g., proteases/inhibitors or immune/defense functions) and male transcripts (e.g., prenyl- dimethylallyl- and geranyl-transferases) associated with reproduction are not as divergent and potentially incompatible as *cis-*only *or transgressive* transcripts.

In hybrids, the pattern of expression in reproductive genes presumably plays an essential role in modulating introgression (Signor and Nuzhdin 2018). Models of postzygotic incompatibilities predict that reproductive genes are affected by the genetic incompatibilities when combining the genomes of the two parental species in one individual (Coyne and Orr 1997; Landry et al. 2005; Masly and Presgraves 2007; Ortiz-Barrientos et al. 2006; Presgraves 2008; Maheshwari and Barbash 2011; Cattani and Presgraves 2012; Turelli et al. 2014; Lopez-Maestre et al. 2017). We have demonstrated that reproductive genes in *Anastrepha* species are in fact associated with transcriptional differentiation having the lesser impact on hybrid fitness, such as *dominant* and *Trans-only*. More interestingly, these results point to *trans*-only regulation and *dominance* as possible mechanisms by which species isolation mechanisms can evolve in the presence of gene flow and divergent selection. The regulatory mechanisms of reproductive genes seem to evolve with *trans*-only divergence while their expression is dominated by one of the species, instead of being associated with the transcriptional misexpression of *transgressive* and *cis*-only transcripts, which are more likely responsible for the reproductive breakdown in hybrids. The widespread pattern of dominance in multiple eukaryotic organisms that can hybridize (McManus et al. 2010; Yoo et al. 2013; Edger et al. 2017; Sanchez-Ramirez et al. 2021; Zhang et al. 2021) further supports this hypothesis.

## Materials and Methods

### Study Population

Crosses were derived from established lab populations of *A. fraterculus* and *A. obliqua* originally collected from fruits of hostplants in Midwest (16^°^ 41′ 58′‘S, 49^o^ 16′ 35′‘W) and Southeastern (22^°^ 01′ 03′‘S, 47^o^ 53′ 27′‘W) regions of Brazil, respectively. Field collected flies were identified using wing, ovipositor, and other morphological markers following identification keys available (Norrbom et al. 2012). These lab populations were maintained in the Population Genetics and Evolution Lab at the Federal University of São Carlos (Brazil) for over 2 years in a controlled environment room at 26 °C (60–90% humidity) and natural photoperiod before the experiment. Mango (*Mangifera indica* L.) fruits were used for oviposition and larval development, whereas adults were fed on a mixture of hydrolyzed protein, vitamins, and sucrose. Populations were maintained by sampling over 100 mating pairs of adults to generate nonoverlapping generations and reduce inbreeding.

Samples from the parental species were derived from individual intraspecific crosses (individually paired *♀ A. fraterculus* x *♂ A. fraterculus* and *♀ A. obliqua* x *♂ A. obliqua*), whereas reciprocal F_1_ hybrids were derived from interspecific crosses (individually paired *♀ A. fraterculus* × *♂ A. obliqua* and *♀ A. obliqua* × *♂ A. fraterculus*). Because the *♀ A. fraterculus* × *♂ A. obliqua* cross only produces female progeny (Santos et al. 2001), our analysis is restricted to three of the four classes of hybrids. To indicate the direction of the cross, we refer to the reciprocal hybrids as OF and FO (F = *A. fraterculus* and O = *A. obliqua*, where the female parent is listed first). Entire male and female reproductive tissues were obtained from 10-day-old mature virgin progeny from each of the crosses. These tissues were chosen because they are likely to express genes that play important roles in species differences as they tend to evolve more rapidly than background genome (Andrés et al. 2013). Groups of five specimens were pooled for each sample, and three replicates were generated per cross, which generated six samples for each parental species and OF hybrids and three for FO hybrids (only females), for a total of 21 samples. All samples were kept on Trizol reagent at −80 °C until RNA extractions.

### RNA Extraction, cDNA Library Construction, and Sequencing

Total RNA was extracted from pooled samples using the Trizol-chloroform protocol (Chomczynski and Mackey 1995). RNA quality was visually inspected by agarose gel electrophoresis and quantified using both a Qubit fluorometer and Nanodrop spectrophotometer. cDNA libraries were created using Illumina TruSeq Stranded mRNA Sample Prep LS Protocol according to the manufacturer's instructions. Libraries were sequenced at the Laboratory of Functional Genomics Applied to Agriculture and Agri-energy, ESALQ-USP, Brazil, using the HiSeq SBS v4 High Output Kit on Illumina platform flow cells with runs of 2 × 100 bp paired-end reads. Illumina's HiSeq Control Software and CASAVA v1.8.2 software (Illumina, Inc.) were used for base calling and sample demultiplexing.

### Sequence Trimming and Assembly

Reads were trimmed for quality and adapter sequences were removed using a minimum quality base of Q = 20 and minimum read length of 50 bp using Trimmomatic (Bolger et al. 2014). Because there is no reference genome for either species, de novo assembly was performed using the software Trinity (Grabherr et al. 2013) with default parameters.

### Improving Transcriptional Assemblies Before Mapping

*De novo* assemblers as implemented in Trinity often generate hundreds of thousands of sequences resulting in a large number of isoforms grouped in components, often with numerous redundancies and chimeric sequences (Yang and Smith 2013). Trinity may generate several sequences that correspond to the same gene (due to alternative splicing) or independent sequences from the same gene as well as combined genes or chimeras. Such complications are maximized when there is increased heterogeneity in the samples, such as when combining samples from different individuals and species (Yang and Smith 2013). To minimize these issues, we performed separated assemblies for male and female reproductive tissues. We then used an assembly cleaning strategy based on the software set Trinity-Bowtie2-RSEM-CD-HIT-EST, to reduce redundant and chimeric sequences, while maximizing the number of truly representative isoforms. This strategy has been empirically demonstrated to show superior results when compared with alternative approaches, improving the accuracy of downstream analyses (Yang and Smith 2013). We used Trinity utilities to filter the assembled transcriptomes by abundance, and mapping reads back to their assemblies using Bowtie2 (Langmead et al. 2009). A read count matrix was generated using RNA-seq by expectation-maximization (RSEM) and only isoforms with the highest percentage of abundance within each component were retained as representative based on read counts after trimmed mean of M-values (TMM) normalization. Then, we reduced the remaining redundancy using CD-HIT-EST with a sequence identity cutoff set to 0.98 to collapse highly similar components within assemblies.

### Allele-specific Expression

Analyses of ASE rely on the identification of the parental origin of reads sequenced in hybrid samples. For this, we identified fixed SNP differences between the two populations. Reads coming from the allele more similar to the reference can potentially map with higher probability or quality than reads coming from the nonreference allele, leading to inaccurate estimates of ASE. To avoid this mapping bias, we accounted for polymorphic variation in the reference using GSNAP (Wu and Nacu 2010), which allows SNP-tolerant alignment in mapping. This method allows SNP information to be introduced into the reference, which allows reads from two different species to be mapped to a “common diploid *Anastrepha* reference” that includes all the divergent SNPs. This SNP-tolerant strategy has been shown to better account for allele mapping bias than alternatives strategies such as SNP masking or reciprocal best hits (Satya et al. 2012).

SNP calling and SNP-tolerant mapping were carried out using modules implemented in the Allim pipeline (Pandey et al. 2013). For this, only one of the initial assemblies (e.g., *A. obliqua*) was retained for each profile, and this assembly was used as a template to generate the “common diploid *Anastrepha* reference” used in further analyses. Allim can automate multiple rounds of SNP identification and GSNAP mapping (with or without the SNP-tolerant option). After mapping the reads from both species to the selected reference assembly, divergent SNPs were identified. Polymorphism information was then used in a subsequent round of SNP-tolerant mapping. SNPs were identified using SAMtools (Li et al. 2009) with the mpileup option, which was then used to call the SNPs through Bayesian inference in bcftools (Li et al. 2009). Because the SNP calling was performed to identify fixed SNPs between species, only SNPs where the species were fixed for different alleles were considered. This allows reads to be unambiguously assigned to one of the parental genotypes. This information was then incorporated into the mapping for an SNP-tolerant alignment via GSNAP. Allim was also used to assess the quality of the common reference by testing for any remaining bias using simulated reads. For this, both references generated were used to simulate the same number of reads for each polymorphic site, and then simulated reads were mapped back to the “common diploid *Anastrepha* genome”.

### Expression Divergence and Allelic Imbalance

We created a read count matrix for the parental species and hybrids using the reads that were unambiguously assigned to their specific parental origin. Count data were rounded to the nearest integer to satisfy the requirements of downstream statistical tests (e.g., negative binomial). The read count matrix was filtered for a minimum count cutoff of 3 cpm for each parental species and hybrids over at least two of three replicates per comparable group. All zero values were then adjusted to one to satisfy the binomial tests used below for *cis* and *trans* classifications because positive integers are required. All expression analyses were performed using the R package *edgeR* (Robinson et al. 2010) after TMM library normalization. Normalized counts were analyzed by generalized linear models accounting for the negative binomial variable of read counts in the case of gene expression as well as binomial variable for ASE in hybrids, followed by analyses of ED between species, modes of inheritance (e.g., *additive*/*nonadditive*) and regulatory divergence (e.g., *cis*/*trans*). An FDR correction (Benjamini and Hochberg 1995) using a global α = 0.05 for multiple comparisons as well as a log_2_-fold-change threshold of > 1.25 was applied to all *P*-values.

### Inheritance Modes of Gene Expression

Modes of inheritance were investigated by comparing global expression levels of a given gene between the parental species *Log*_2_*(P*_1_*/P*_2_*)* and between hybrids and each parental species: *Log*_2_*(P*_1_*/F*_1_*)* and *Log*_2_*(P*_2_*/F*_1_*)* while ignoring allelic information (total gene expression levels in hybrids reflect the sum of reads mapped to both parental alleles). A negative-binomial GLM analysis and implemented in edgeR software was used to evaluate pairwise comparisons of gene expression between hybrids and their parental species. Based on these comparisons, each transcript was classified according to a commonly used system to describe *additive* and *nonadditive* modes of gene expression (fig. 1*a*) (McManus et al. 2010; Bell et al. 2013). Nondifferential transcripts between parents and hybrids were classified as *conserved*. Transcripts for which expression in the hybrid is not significantly different from one of the parents were considered as *dominant* for that parent. Transcripts for which hybrid expression was not similar to either parent but is within the parental range were classified as *additive*, whereas transcripts for which expression in the hybrid is either above or below parental range were considered as *transgressive* (*overdominant* and *underdominant*, respectively).

### Cis/Trans Regulatory Divergence

Gene expression can be regulated by *cis*-acting or *trans*-acting effects (fig. 1*b*). The contribution of regulatory effects on gene expression was investigated by comparing the extent of allele imbalance in hybrids (i.e., the relative expression of allele *A*_1_ derived from species 1 and allele *A*_2_ from species 2):


$$R_{h}\;=\;\mathrm{Lo}g_{2}(A_{1(h)}/A_{2(h)})$$

and the relative expression of these alleles when homozygous in the parental species (Haerty and Singh 2006; McManus et al. 2010; Bell et al. 2013):


$$R_{p}\;=\;\mathrm{Lo}g_{2}(P_{1}/P_{2})$$

In the genetic regulatory background of the hybrid, both parental alleles are exposed to the same *trans*-effects, which means that any allele imbalance will be the result of a *cis*-regulatory effect (hence, *R_h_* represents the *cis*-regulatory effect, where the allele present at a locus is responsible for variation in its own expression). Consequently, any deviation on the degree of ED between parents and the degree of allele imbalance in hybrids indicates the occurrence of *trans*-regulatory effects. Hence, the *trans* effect is given by:


$$T=R_{p}-\;R_{h}$$

A negative-binomial GLM analysis was used to evaluate significant *cis*-effects, based on allelic imbalance in the hybrids. *Trans*-effects were identified through a binomial GLM comparing the ratios of ASE between hybrids (*R_h_*) and parents (*R_p_*). Thus, nondifferential ratios are evidence of *cis*-only regulatory divergence, whereas any significant deviation between ratios is evidence of additional *trans*-effects favoring the expression of one allele.

Based on the extent and direction of expression change, we classified transcripts in seven different patterns. Transcripts were classified as *conserved* if there was no significant differential expression between parents, between alleles in hybrids, and between their ratios. Transcripts show *cis*-only regulation when there is significant differential expression between parents, and that pattern is retained for the alleles in hybrids, but no significant differences between their ratios. Transcripts show *trans*-only regulation when there is significant differential expression between parents, but not between alleles in hybrids, with significant differences between their ratios. Transcripts may also show a mixture of these, with significant differential expression between parents, between alleles in hybrids, and significant differences between their ratios. In the case where there is an expression difference between parental and hybrid alleles that goes in direction of the same allele, transcripts are classified as *cis* + *trans*, whereas when the expression differences are biased toward different alleles, transcripts are classified as *cis x trans*. Transcripts are classified as *compensatory* when there is no significant differential expression between parents, but there are significant differences between alleles in hybrids and significant differences between their ratios. In this case, opposite changes between *cis* and *trans*-effects compensate each other, resulting in no expression differences between species. Finally, transcripts are classified as *ambiguous* when expression patterns did not follow any clear expectations according to these criteria.

### The cis Index

To characterize the overall pattern of regulation, we quantified the relative effect of *cis*-acting versus *trans*-acting mechanisms for each transcript. For this, we created an index that measures the size of the *cis* component relative to the total *cis* and *trans* effects:


$$I_{cis}=|R_{h}|/(\left|R_{h}|+|T\right|)$$

This index was used to investigate the relative role of these mechanisms concerning expression inheritance and molecular and EDs.

### Identification of Potential X-linked Transcripts

To evaluate the potential contribution of X-linked transcripts to patterns of regulatory divergence and gene expression inheritance observed in males in the absence of a reference genome for *Anastrepha* species, we inferred potential X linkage from the expression data. Transcripts were designated as putatively X-linked if they showed significant AI with the complete absence of read counts from the *A. fraterculus* allele in hybrid males (because all X-linked transcripts in males are inherited from the *A. obliqua* parental line). Because these transcripts cannot be unambiguously assigned to the X chromosome, the counts generated by this method provide an upper-limit estimate to the number of X-linked transcripts that could potentially be contributing to the patterns observed in each category of transcripts.

### Molecular Divergence between Species

We estimated molecular divergence between species using the ASE dataset. For this, an additional SNP-tolerant mapping via GSNAP using only parental samples was performed. SNPs between species were identified using SAMtools (Li et al. 2009) with the *mpileup* option, and then allele frequencies were obtained using PoPoolation II software with a minimum quality base of Q = 20 and minimum allele frequency MAF = 2%. We then calculated the interspecific differentiation index (*d*) as the absolute difference in allele frequencies between species (|*frat—obliq*|) for each SNP and then estimated the molecular divergence as the percentage of fixed variation per transcript (*d*_*xy*_ = number of fixed SNPs/total number of SNPs). Then, protein coding sequence (CDS) sequences were predicted using the TransDecoder software (http://transdecoder.github.io), and evolutionary rates were estimated with the KaKsCalculator program (Zhang et al. 2006) The estimated evolutionary rate between species was further used to investigate its relationship with the expression inheritance and regulatory divergence data.

### Molecular Divergence and Regulatory Dynamics

To investigate potential associations between different categories of expression and molecular divergence, we implemented a GLM framework comparing variables of interest among categories/profiles. A comparative matrix containing all transcripts, including ED, inheritance modes, regulatory divergence categories, *cis* index of gene regulation (proportion of gene expression relative to *trans* effect for each transcript), and Ka/Ks estimates between species was generated. GLM analyses were performed using sex, direction of the cross (OF and FO) categories of regulatory divergence, inheritance modes, and tissues as independent variables, whereas both the number of transcripts detected and the ED were considered dependent variables. Then, the response variables were compared across categories of inheritance, reciprocal hybrids (OF and FO). Because postzygotic incompatibilities follow Haldane's rule, hybrid males are not viable when crossing *A. obliqua* females with *A. fraterculus* males (Selivon et al. 1999; Santos et al. 2001; Rull et al. 2018). Due to the absence of males in one direction of the cross, we implemented an incomplete GLM model, which allowed us to estimate both the sex and direction of the cross simultaneously. With this analysis, our results regarding sex differences rely on one direction of the cross, whereas the effect of the cross direction depends only on female data but prevent us from estimating interaction effects involving both sex and the direction of the cross.

Because the number of transcripts detected under each category depends on the transcriptome length (number of assembled transcripts), we normalized all transcript counts to the number of assembled contigs per library. Because several variables are calculated as proportions (e.g., *cis* index, Ka/Ks, normalized number of transcripts), GLM analyses were performed after square-root transformation.

### Transcriptional Annotation and Functional Analysis

Assembled transcriptomes were annotated using the Trinotate framework (http://trinotate.github.io). For this, we used predicted CDS regions from TransDecoder to perform homology searches against known sequence databases (e.g., SWISSPROT) using the programs BLASTX (transcripts) and BLASTP (predicted CDS) (Camacho et al. 2009). HMMER software was used to identify conserved functional domains (Wheeler and Eddy 2013) against the PFAM-A database. rRNA sequences were identified in transcripts using the RNAMMER tool (Lagesen et al. 2007). Finally, the signalP software (Almagro Armenteros et al. 2019) was used to identify signal peptides (secretion signals), whereas the tmHMM tool was used to identify contigs by predicting transmembrane domains (Krogh et al. 2001). All these searches were integrated into a Trinotate SQLite database to produce the final annotation file. Overrepresentation of specific categories of biological functions was investigated for transcripts following inheritance modes of gene expression and regulatory divergence using the GOseq R package framework (Young et al. 2010).

## Supplementary Material

evad071_Supplementary_Data

## Data Availability

All RNAs-seq reads underlying this article have been deposited in the Sequence Read Archive at NCBI under BioProject ID PRJNA934045.
