# Supplementary material for: Transcriptional Misexpression in Hybrids between Species Linked by Gene Flow Is Associated With Patterns of Sequence Divergence
Source: Genome Biol Evol. 2023 May 8;15(5):evad071. doi: 10.1093/gbe/evad071 (PMC10195090; doi:10.1093/gbe/evad071)
Supplement: evad071_Supplementary_Data [file evad071_supplementary_data.docx]

**Transcriptional misexpression in hybrids between species linked by gene flow is associated with patterns of sequence divergence**

**Supplementary Material**

**TABLES**

**Table S1.** Sample profiles used for RNA-Seq analysis. Each replicate corresponds to a pool of five individuals.

| *Sample* | *Sex* | *Replicates* | *Mean read pairs ± Standard Error* |
| --- | --- | --- | --- |
| *A. fraterculus* | |  |  |
|  | ♀ | 3 | 13883827 ± 319748 |
|  | ♂ | 3 | 13249470 ± 719148 |
| *A. obliqua* | |  |  |
|  | ♀ | 3 | 13132508 ± 1307881 |
|  | ♂ | 3 | 12357116 ± 296177 |
| *Hybrid: ♀ frat x ♂ obliq* | | |  |
|  | ♀ (only) | 3 | 27316867 ± 4083976 |
| *Hybrid: ♀ obliq x ♂ frat* | | |  |
|  | ♀ | 3 | 12418845 ± 464623 |
|  | ♂ | 3 | 13713823 ± 709307 |

**Table S2.** **Percentage of reads classified according to their parental alleles in hybrids between *A. fraterculus* and *A. obliqua*.** Hybrids exhibited a nearly 1:1 ratio of mapping reads based on their parental origins.

| *Sample* |  | *Sex* | *Total transcripts* | *frat allele* | *obliq allele* |
| --- | --- | --- | --- | --- | --- |
| *Hybrid: ♀ frat x ♂ obliq* | | |  |  |  |
|  |  | ♀ (only) | 9709 | 48.98% | 51.02% |
| *Hybrid: ♀ obliq x ♂ frat* | | |  |  |  |
|  |  | ♀ | 9709 | 51.33% | 48.67% |
|  |  | ♂ | 10628 | 51.31% | 48.69% |

**Table S3.** **Summary of transcripts with significant expression divergence (ED) between parental species.** The percentage of significant ED transcripts is indicated between brackets.

| *Sex* | *Females* | *Males* |
| --- | --- | --- |
| Total transcripts | 9709 | 10628 |
| **Expression divergence** |  |  |
| FDR *α* = 0.05 | 3513 (36%) | 2360 (22%) |
| FDR *α* = 0.05 and log_2_FC > 1.25 | 2248 (23%) | 1177 (11%) |

**Table S4.** **Summary of transcripts with significant allelic imbalance (i.e., allele specific expression: ASE) and their classifications according to inheritance modes and regulatory mechanisms responsible for divergence.** The percentage of detected transcripts is indicated in parentheses for each category.

| ***Sex*** | ***Females*** | ***Females*** | ***Males*** |
| --- | --- | --- | --- |
| **Cross** | *♀ f* × *♂ o* | *♀ o* × *♂ f* | *♀ o* × *♂ f* |
| Total transcripts | 9709 | 9709 | 10628 |
| **ASE** |  |  |  |
| FDR *α* = 0.05 | 1919 (19.8%) | 1560 (16.1%) | 1765 (16.6%) |
| FDR *α* = 0.05 and log_2_FC > 1.25 | 1113 (11.5%) | 943 (9.7%) | 1437 (13.5%) |
| **Inheritance modes** |  |  |  |
| *Additive* | 30 (0.3%) | 67 (0.7%) | 41 (0.4%) |
| *Dominant-frat* | 1330 (13.7%) | 986 (10.2%) | 407 (3.8%) |
| *Dominant-obliq* | 99 (1%) | 152 (1.6%) | 189 (1.8%) |
| *Overdominant* | 33 (0.3%) | 35 (0.4%) | 68 (0.6%) |
| *Underdominant* | 11 (0.1%) | 11 (0.1%) | 25 (0.2%) |
| *Conserved* | 8206 (84.5%) | 8458 (87.1%) | 9898 (93.1%) |
| **Regulatory divergence** |  |  |  |
| *Cis-only* | 31 (0.3%) | 18 (0.2%) | 26 (0.2%) |
| *Trans-only* | 1300 (13.4%) | 1426 (14.7%) | 542 (5.1%) |
| *Cis + Trans* | 4 (0.04%) | 10 (0.1%) | 1 (0.01%) |
| *Cis * Trans* | 500 (5.1%) | 437 (4.5%) | 478 (4.5%) |
| *Compensatory* | 381 (3.9%) | 326 (3.4%) | 681 (6.4%) |
| *Conserved* | 5390 (55.5%) | 5321 (54.8%) | 6522 (61.4%) |
| *Ambiguous* | 2103 (21.7%) | 2171 (22.4%) | 2378 (22.4%) |

**Table S5. Generalized linear model analysis of the number and expression of transcripts across categories of inheritance and regulatory divergence.** Both the number of transcripts and their expression were compared. Data for the number of transcripts were normalized across libraries and then square root transformed. Significant *P*-values are highlighted in bold.

| *Effect* | *Number of transcripts* | | | |  | *Expression divergence* | | | |
| --- | --- | --- | --- | --- | --- | --- | --- | --- | --- |
|  | *Df* | *Resid. Df* | *F* | *P* |  | *Df* | *Resid. Df* | *F* | *P* |
| ***Inheritance modes*** |  |  |  |  |  |  |  |  |  |
| Category | 4 | 10 | 77.1 | **< 0.001** |  | 4 | 3479 | 12.2 | **< 0.001** |
| Sex | 1 | 9 | 3.4 | 0.123 |  | 1 | 3478 | 69.0 | **< 0.001** |
| Category * Sex | 4 | 5 | 8.7 | **0.018** |  | 4 | 3474 | 3.5 | **0.007** |
|  |  |  |  |  |  |  |  |  |  |
| ***Regulatory divergence*** |  |  |  |  |  |  |  |  |  |
| Category | 4 | 10 | 446.6 | **< 0.001** |  | 4 | 6156 | 133.0 | **< 0.001** |
| Sex | 1 | 9 | 16.1 | **0.010** |  | 1 | 6155 | 24.7 | **< 0.001** |
| Category * Sex | 4 | 5 | 38.6 | **< 0.001** |  | 4 | 6151 | 17.6 | **< 0.001** |

**Table S6. Generalized linear model analysis of the proportion of expression divergence due to *cis* effects relative to *trans* effects.** The *cis* index was estimated per transcript and compared across categories of inheritance and sex. Data for the *cis* component were normalized following square root transformation. Significant *P*-values are highlighted in bold.

| *Effect* | *Df* | *Resid. Df* | *F* | *P* |
| --- | --- | --- | --- | --- |
| Inheritance mode | 4 | 3479 | 86.8 | **< 0.001** |
| Sex | 1 | 3478 | 81.6 | **< 0.001** |
| Inheritance * Sex | 4 | 3474 | 7.5 | **< 0.001** |

**Table S7. Relative proportions of genes following categories of gene expression inheritance and regulatory divergence.** The percentage of genes found under each category is shown for hybrids between species with different levels of phylogenetic distance and for intraspecific comparisons.

| **Inter- vs Intraspecifc** | **Species** | **Cis-only** | **Trans-only** | **Cis-Trans * Interactions** | **Additive** | **Dominant** | **Transgressive** | **Reference** |
| --- | --- | --- | --- | --- | --- | --- | --- | --- |
| Interspecific | *Drosophila simulans –*  *D. sechellia* | NA | NA | 10.1% | 8% | 35% | 20% | Coolon et al. 2014 |
| Interspecific | *D. melanogaster –*  *D. simulans* | NA | NA | 20% | 5% | 18% | 80% | Coolon et al. 2014 |
| Interspecific | *D. melanogaster –*  *D. sechellia* | 12% | 28% | 35% | 16% | 49% | 35% | McManus. 2010 |
| Interspecific | *D. melanogaster –*  *D. simulans* | NA | NA | NA | 1.4% | 10.9% | 69.30% | Ranz et al 2004 |
| Interspecific | *Canordhabditis briggsae – C. nogoni* | Males: 18% Females: 14% | Males: 17% Females: 12% | Males:  27% Females: 31% | Males:  8% Females: 8% | Males: 30% Females: 23% | Males:  26%  Females:  55% | Sanchez-Ramirez et al. 2021 |
| Interspecific | *Camellia chekiangoliosa – C. azalea* | 3.30% | 9% | 2.50% | 4.60% | 65.50% | 31% | Zhang et al. 2019 |
| Interspecific | *Camellia azalea –*  *C. amplexicaulis* | 5.40% | 3.30% | 2% | 1.50% | 61.30% | 37.30% | Zhang et al. 2019 |
| Intraspecific | *D. melanogaster* | NA | NA | 10% | 7% | 43% | 14% | Coolon et al. 2014 |
| Intraspecific | *Cirsium arvense* | 2% | 3% | 52% | 6% | 38% | 29% | Bell et al. 2013 |
| Intraspecific | *D. pseudoobscura* | 7.90% | 16.90% | 5.50% | 8% | 40.90% | 17.30% | Suvorov et al. 2013 |

* Cis-Trans interactions includes Cis x Trans and compensatory categories.

**FIGURES**


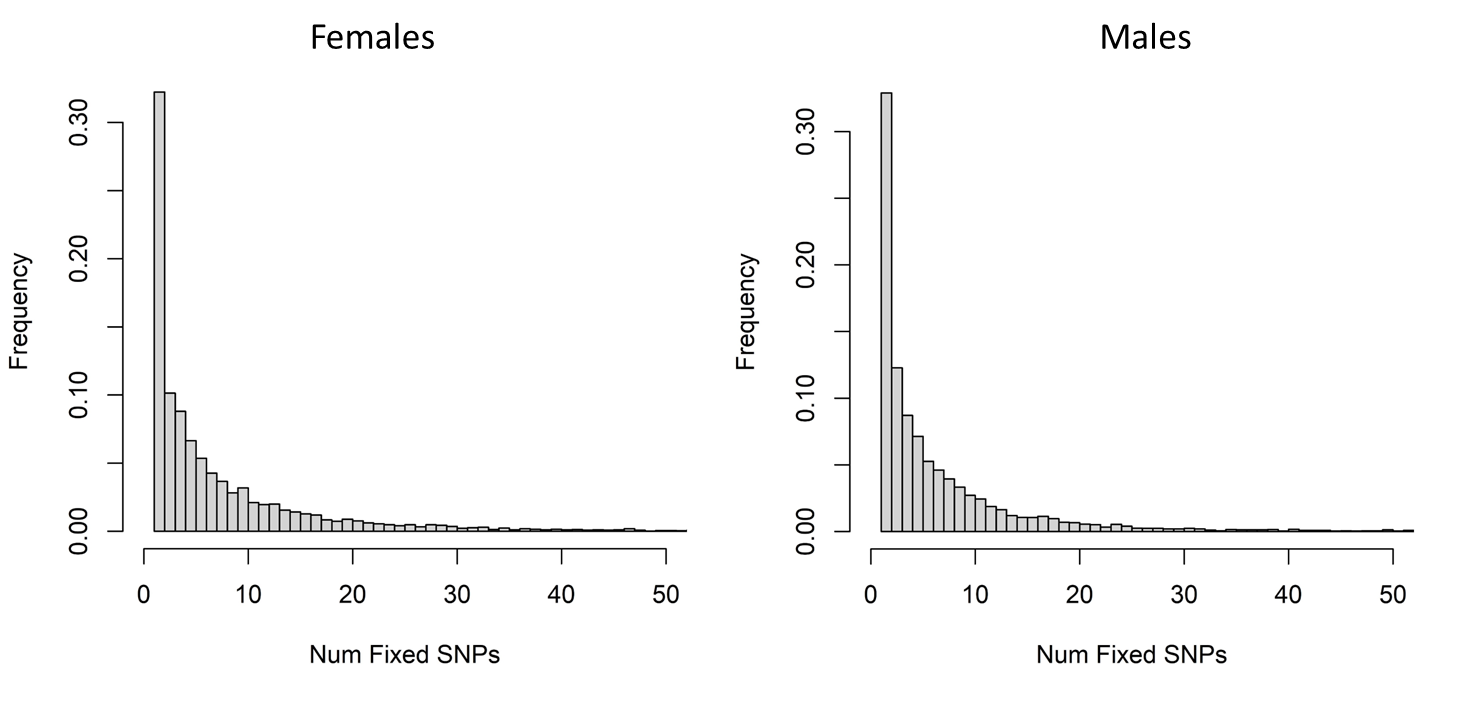
**Figure S1. Frequency distribution of fixed SNPs per transcript between *A. fraterculus* and *A. obliqua*.** The number of fixed SNPs followed similar distributions in male' and female's reproductive transcriptomes. On average, there were 8.15 SNPs per transcript in females and 7.20 SNPs in males, and a median of 4 SNPs per transcript in both cases.

**
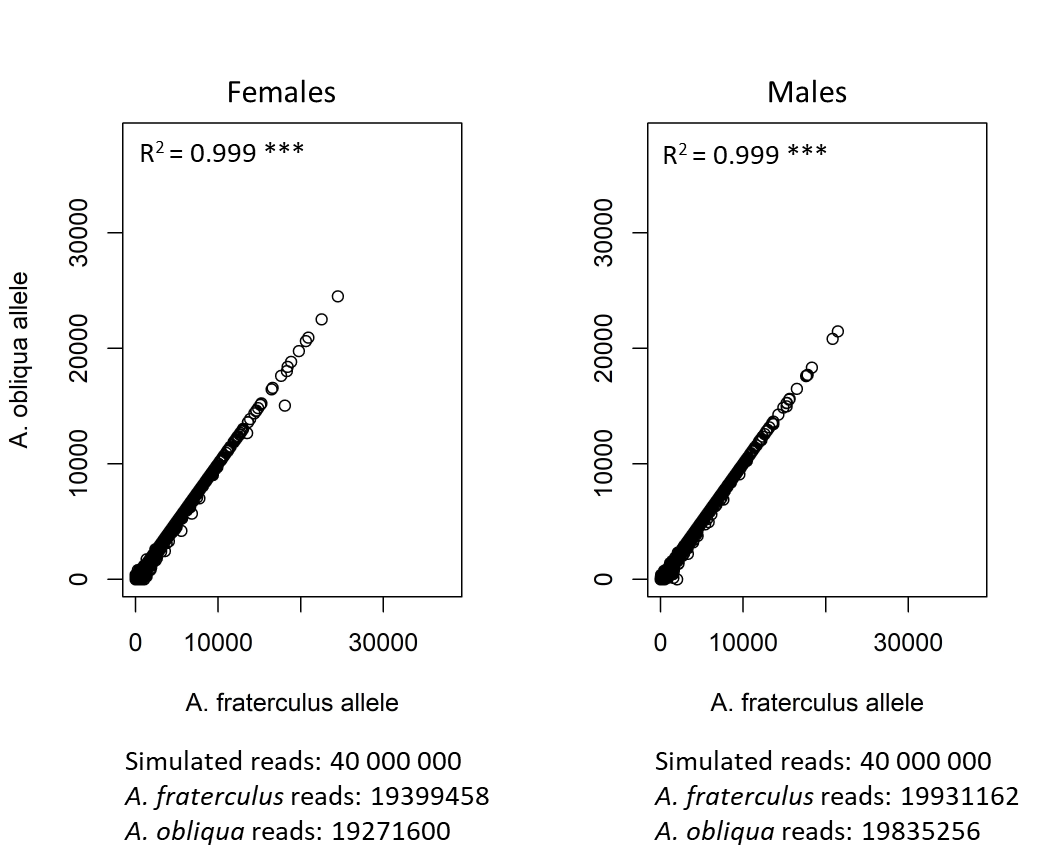
**

**Figure S2. Allim results show a simulation of reads from the** **"diploid *Anastrepha* genome".** Allim software was used to assess the quality of the common reference by testing for any remaining bias using simulated reads. Both references were used to simulate the same number of reads for each polymorphic site, and then simulated reads were mapped back to the "common diploid *Anastrepha* genome". As expected in the absence of mapping bias, transcripts in all samples followed an expression ratio of nearly 1:1.


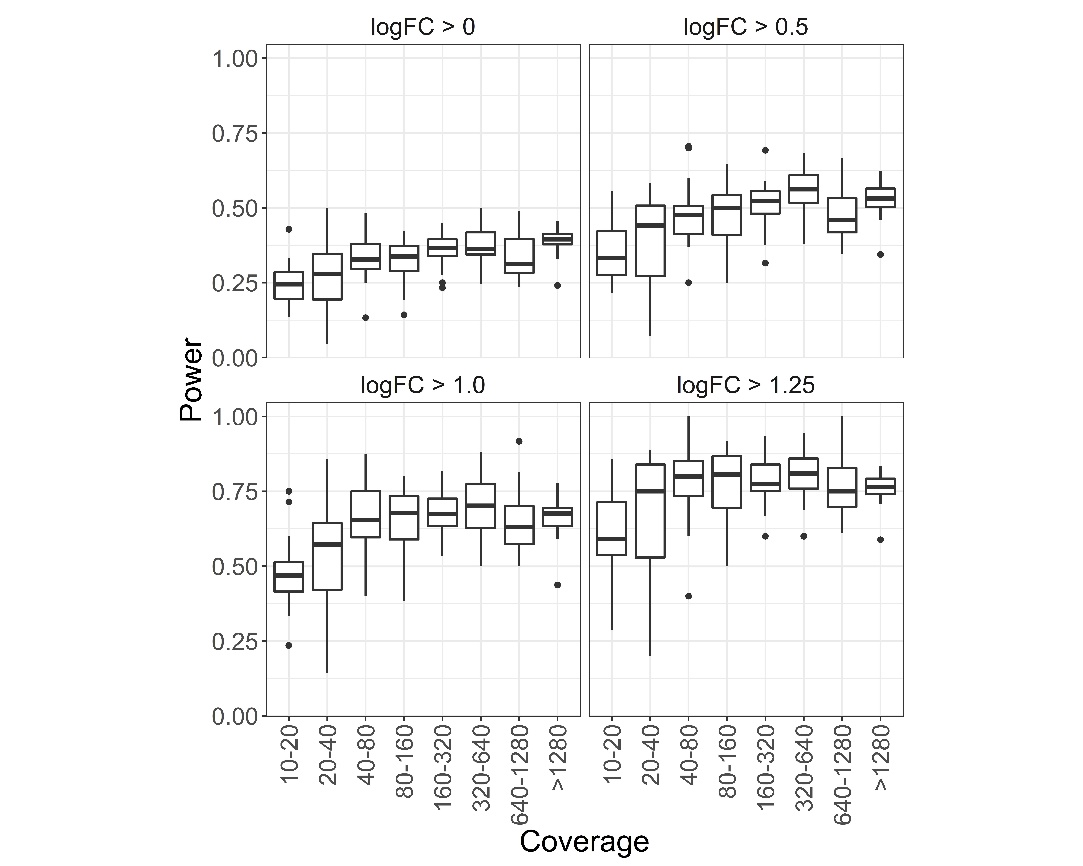


**Figure S3. Power analysis for detection of allele-specific expression (ASE).** The R package "*Proper*" (Wu et al. 2015) was used to perform 20 simulations of RNASeq data. Parameters used for simulations were defined based on the ASE data from hybrids between *A. fraterculus* and *A. obliqua*. These parameters involved an average coverage of 4.5 Million reads per group, 9709 transcripts, and three biological replicates per treatment with high dispersion. The power to detect significant differential expression was then estimated using the same FDR of 0.05 and four different thresholds for differential gene expression (log_2_FC > 0, log_2_FC > 0.5, log_2_FC > 1.0, and log_2_FC = 1.25). The power to detect significant differentially expressed transcripts with these parameters increases with the established threshold and becomes closer to the canonical 80% for the used threshold of 1.25.

**Figure S4. Expression divergence and allelic imbalance in hybrids between *A. fraterculus* and *A obliqua*. a)** Boxplots show the distribution of transcripts with expression divergence (*log_2_FC*) between the parental species for each reproductive tissue. **b)** Boxplots show the distribution of transcripts with the allelic imbalance (*log_2_FC*) between alleles in F_1_ hybrids for each direction of the cross (*e.g.*, OF vs FO, see methods).

**
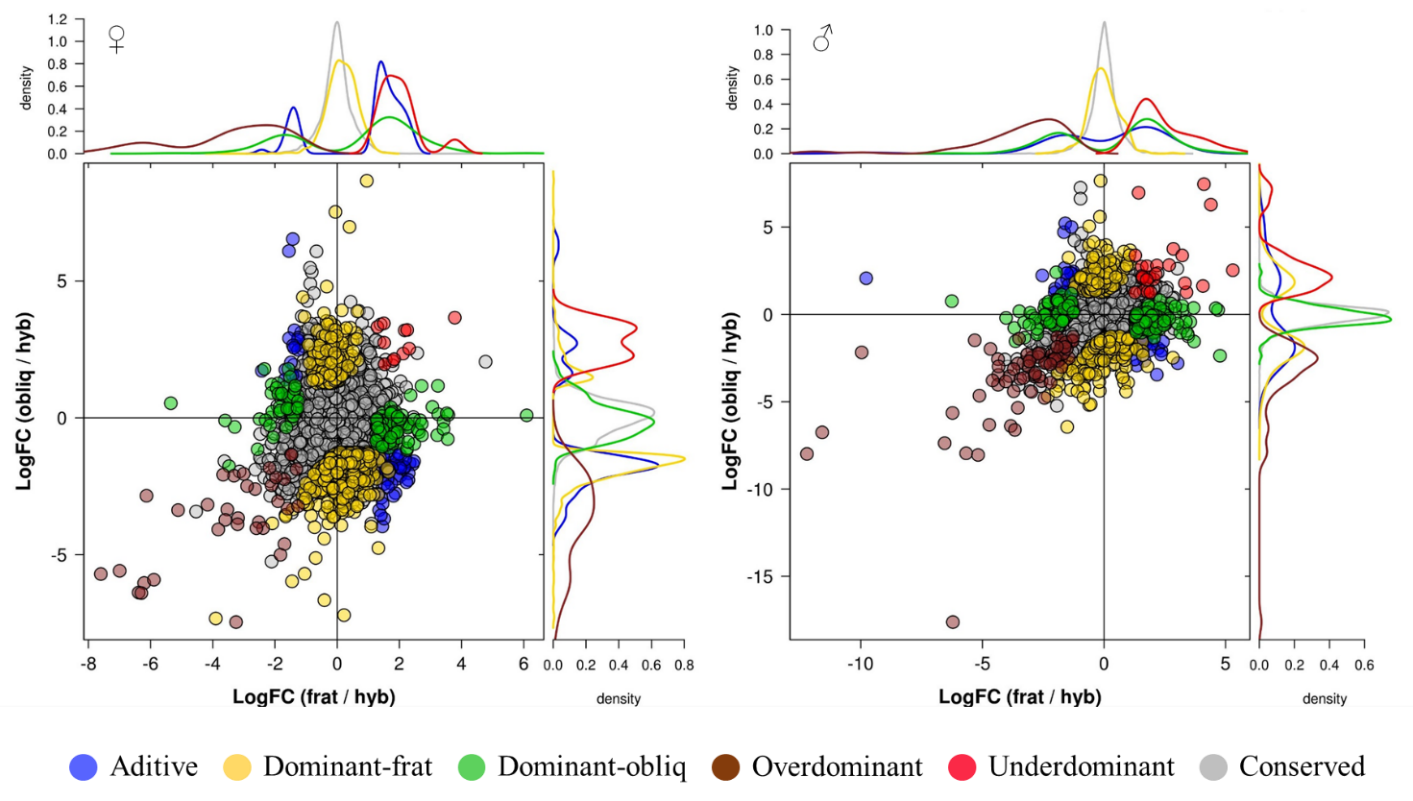
Figure S5.** **Modes of expression inheritance in hybrids between *A. fraterculus* and *A. obliqua***. Scatterplots compare the relative expression of either parent (*A. fraterculus* / *A. obliqua*) and the hybrid [*log_2_FC(frat*/*hyb*) and *log_2_FC(obliq*/*hyb*)], where hybrid expression is the sum of expression of the two alleles in hybrid (*hyb = hyb-frat + hyb-obliq*). These results were used to sort genes into categories based on their inheritance modes.

**
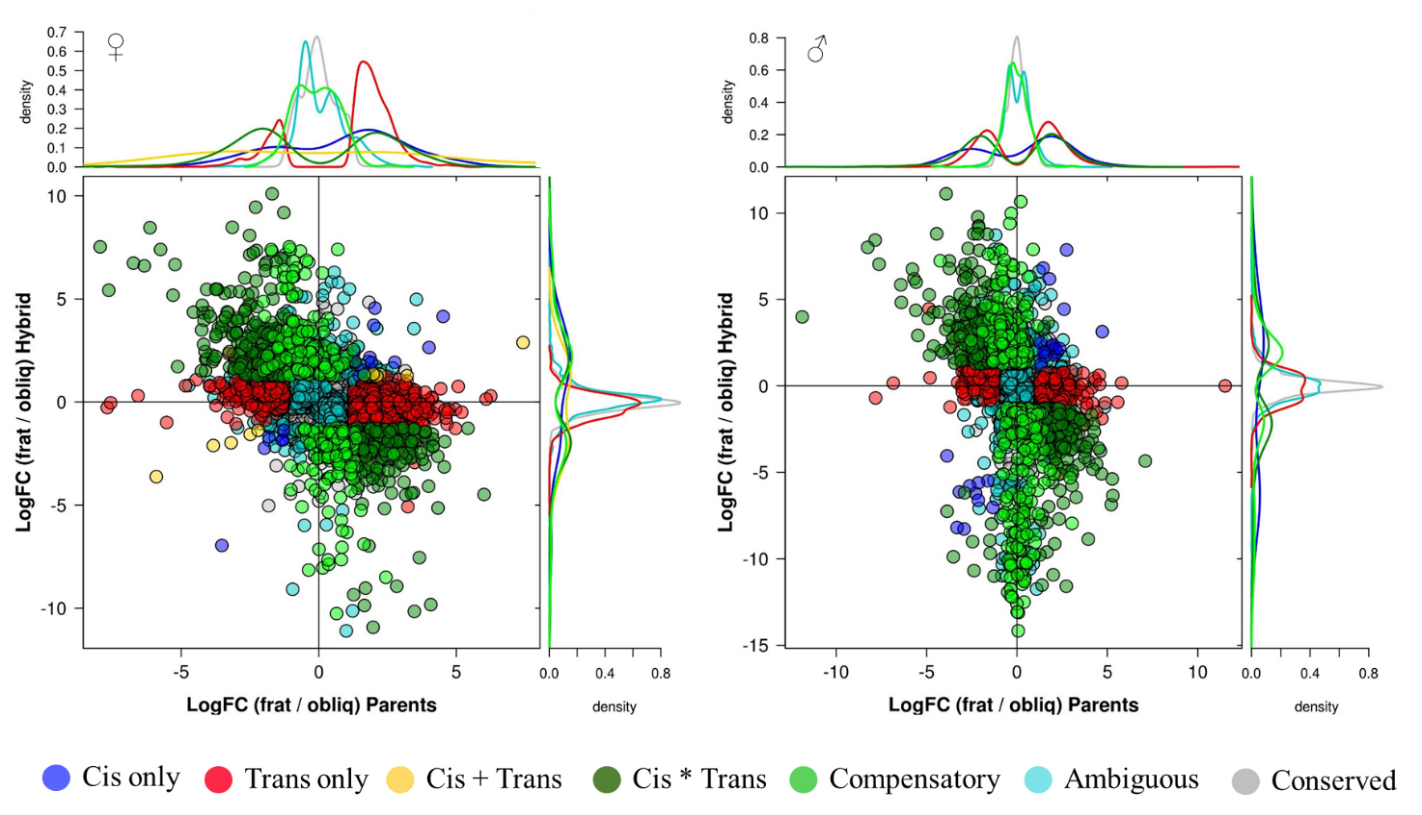
Figure S6.** **Regulatory divergence due to combinations of *cis* and *trans* effects between *A. fraterculus* and *A. obliqua***. Scatterplots compare relative allelic expression levels between parental species [*log_2_FC(frat/obliq)*] and between alleles in hybrids [*log_2_FC(hyb_frat + hyb_obliq)*]. These results were used to sort genes into categories based on their mechanism of regulatory divergence.
